# Perspective: Is the Response of Human Energy Expenditure to Increased Physical Activity Additive or Constrained?

**DOI:** 10.1016/j.advnut.2023.02.003

**Published:** 2023-02-23

**Authors:** Javier T. Gonzalez, Alan M. Batterham, Greg Atkinson, Dylan Thompson

**Affiliations:** 1 Department for Health, University of Bath, Bath, United Kingdom; 2 Centre for Nutrition, Exercise, and Metabolism, University of Bath, Bath, United Kingdom; 3 Professor Emeritus, School of Health and Life Sciences, Teesside University, Middlesborough, United Kingdom; 4 Research Institute for Sport and Exercise Sciences, Liverpool John Moores University, Liverpool, United Kingdom

**Keywords:** energy expenditure, metabolism, energy balance, physical activity, exercise

## Abstract

The idea that increasing physical activity directly adds to TEE in humans (additive model) has been challenged by the energy constrained hypothesis (constrained model). This model proposes that increased physical activity decreases other components of metabolism to constrain TEE. There is a logical evolutionary argument for trade-offs in metabolism, but, to date, evidence supporting constraint is subject to several limitations, including cross-sectional and correlational studies with potential methodological issues from extreme differences in body size/composition and lifestyle, potential statistical issues such as regression dilution and spurious correlations, and conclusions drawn from deductive inference rather than direct observation of compensation. Addressing these limitations in future studies, ideally, randomized controlled trials should improve the accuracy of models of human energy expenditure. The available evidence indicates that in many scenarios, the effect of increasing physical activity on TEE will be mostly additive although some energy appears to “go missing” and is currently unaccounted for. The degree of energy balance could moderate this effect even further.

## Statements of Significance

Current evidence for the constrained energy hypothesis is subject to limitations, including methodological, statistical, and deductive inference. Suitably powered randomized controlled trials with measures of energy balance components are needed to better elucidate whether physical activity is additive or constrained.

## Introduction

The constrained energy expenditure hypothesis challenges the notion that increases in AEE add to TEE. This hypothesis was first proposed by Herman Pontzer [1], and the overarching premise is conceptualized with the following statement from his recent book:

*“The bottom line is that your daily* (physical) *activity level has almost no bearing on the number of calories that you burn each day” (p103)* [2].

The potential controversy of this topic has been briefly highlighted [3]. If this hypothesis is true, it has profound ramifications for scientific understanding of energy balance and prevention and management of obesity. The potential to manipulate energy expenditure with physical activity and/or calculate energy requirements for the population would also be severely challenged. The aim of this review is to provide an independent appraisal of the current evidence used to support the constrained energy expenditure hypothesis and to highlight future directions for research.

## Human energy expenditure is comprised of multiple components

**FIGURE 1:**
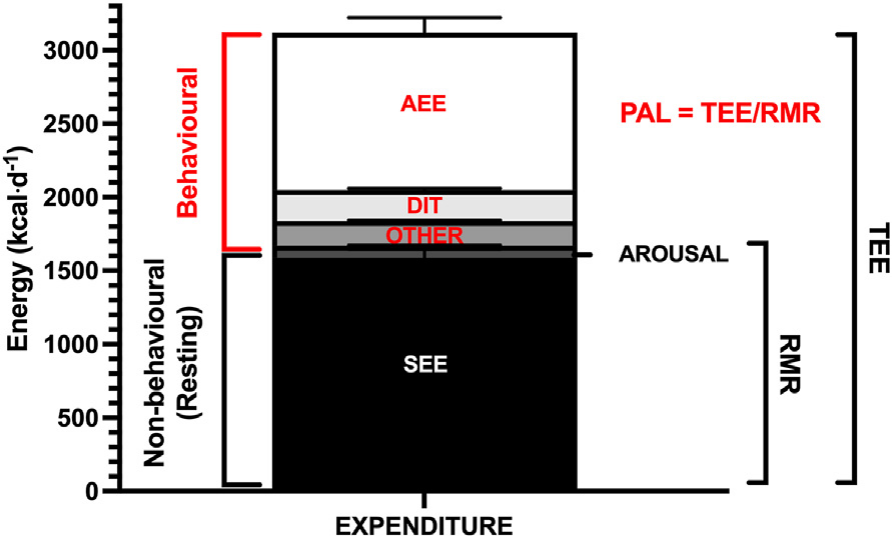
Components of energy expenditure in 75 healthy adults. Components in pink are primarily behavioral. Components in black are primarily nonbehavioral. Data adapted from Chrzanowski-Smith et al. [41]. PAL is calculated by dividing RMR (the sum of SEE and arousal) by TEE. DIT, diet-induced thermogenesis; SEE, sleeping energy expenditure.

Human TEE is the energy cost of all metabolic processes and is comprised of several components (Figure 1). The primarily nonbehavioral components include:

1) sleeping metabolic rate (SMR);
2) arousal (when awake) [4];and
3) cold- and heat-induced energy expenditure (which increases TEE by 3 to 7% with typical changes in ambient temperature [5]).

Behavioral components include diet-induced thermogenesis (DIT; a.k.a., the TEF, or specific dynamic action of food), representing increased metabolic rate due to digestion, absorption, and metabolism of ingested energy [6]. Although this does have a nonbehavioral component, most of the variance in diet-induced thermogenesis is explained by the amount and type of energy consumed and thus arises as a consequence of eating behaviors [6]. Finally, AEE is the increase in energy expenditure with skeletal muscle force production [7]. Exercise energy expenditure (EXEE) is a subcomponent of AEE that is planned or structured and thus is defined by the person’s intention, with nonexercize activity thermogenesis (NEAT) comprising the remaining fraction of AEE. Again, whereas some variance in AEE is from nonbehavioral factors, such as the efficiency of movement, most variance is explained by behavioral factors, such as the magnitude and nature of activity [8]. Because the absolute energy cost of movement varies according to body size, the level of physical activity is often expressed as TEE divided by RMR, known as the PAL [9].

## What is the constrained energy expenditure hypothesis?

The constrained energy expenditure model proposes that:

*“The human body adapts dynamically to maintain TEE within a narrow physiological range. Rather than increasing with physical activity in a dose-dependent manner, experimental and ecological evidence suggests the hypothesis that TEE is a relatively constrained product of our evolved physiology”* [10].

**FIGURE 2:**
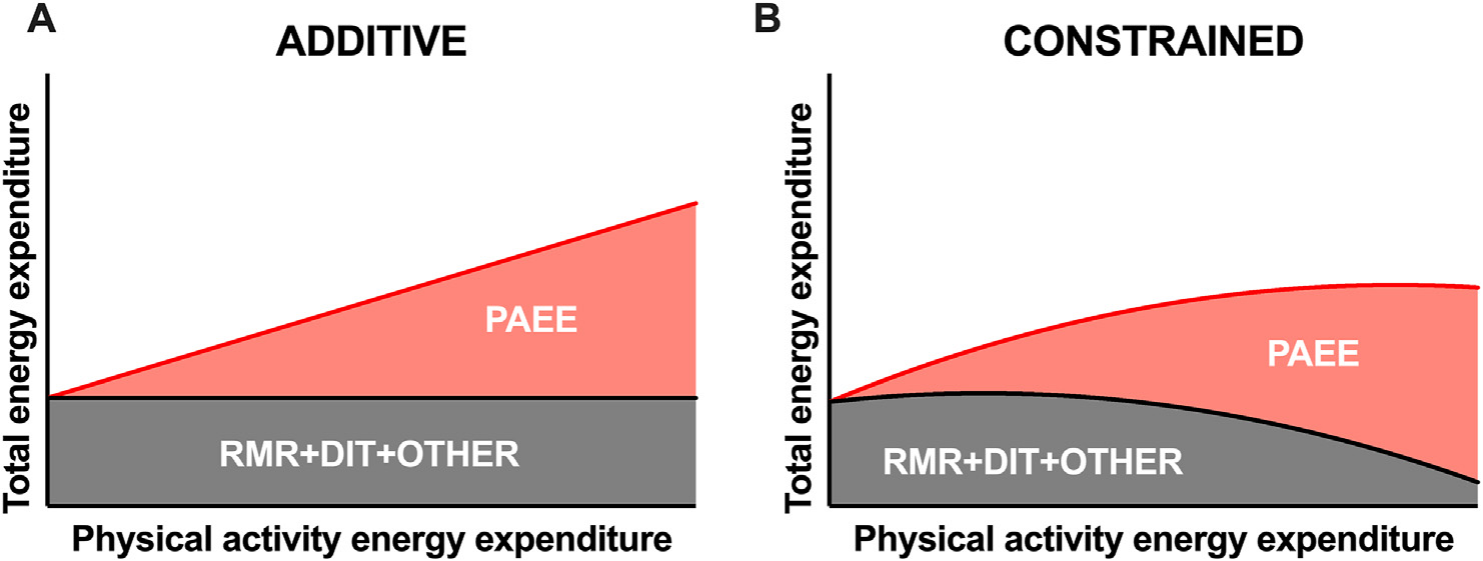
Additive and constrained energy expenditure models as proposed by Pontzer. Adapted from [11]. DIT, diet-induced thermogenesis; PAEE, physical AEE. ADJ, adjusted for body composition and/or age.

In other words, in contrast to the notion of physical activity *directly adding* to TEE (Figure 2A), the energy constrained hypothesis proposes a compensatory decrease in other components of energy expenditure, such that TEE remains relatively constant (Figure 2B).

**FIGURE 3:**
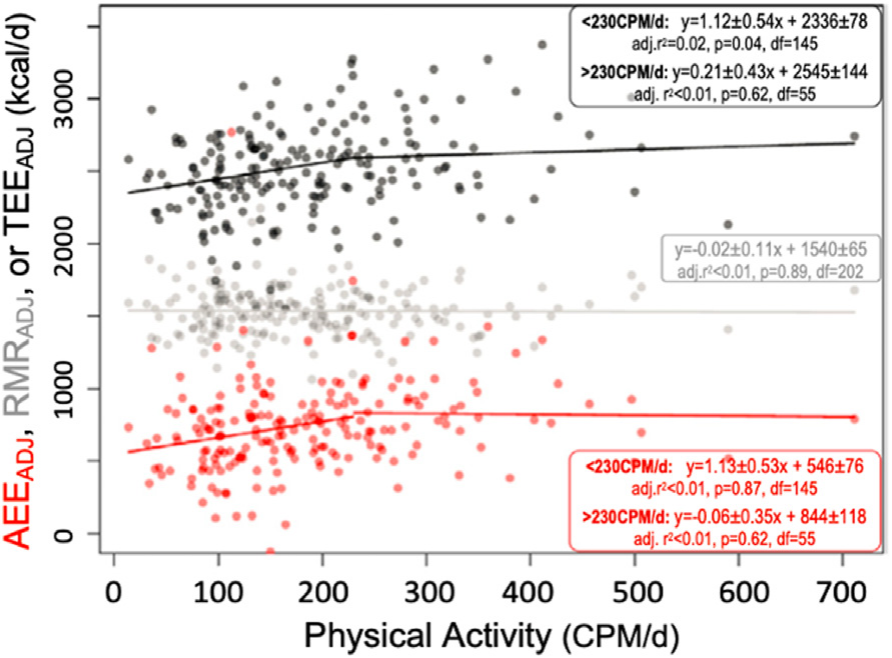
Adjusted TEE (from DLW), RMR, and AEE in relation to increasing PALs estimated by accelerometry. Reprinted from [11].

Initial support for the constrained energy expenditure hypothesis came from a cross-sectional study using DLW to estimate TEE in 30 Hadza (a population of hunter-gatherers) and compared these data with measurements in “Western” and “Farming” populations [1]. PAL was derived using TEE minus BMR, which for the Hadza, was predicted from equations. In contrast to the authors’ expectations, after adjusting for FFM and age, TEE was not significantly different between Hadza compared with Western comparators [1]. PAL was ∼6% (women) and 25% (men) higher in the Hadza compared with Western population, and it was deduced that the Hadza must therefore spend a smaller proportion of TEE on BMR—with the inference that BMR is adjusted downwards when physical activity is high to constrain TEE. This initial report was followed up by a larger study across 332 men and women from 5 diverse locations and populations [11]. This study used DLW over 7 d, measured RMR, and assessed physical activity using a hip-mounted tri-axial accelerometer (Actical, Phillips Ltd) over 6 d (≥62% of a day, and ≥4 d of data were used) [11]. Across the whole sample, a positive linear relationship was reported between accelerometer counts and TEE up to a proposed threshold of ∼230 counts per minute per day (CPM/d), but above this level, additional accelerometer counts did not predict TEE (Figure 3). Unlike findings from the earlier study that predicted RMR [1], there was no evidence for any effect of measured RMR on TEE when measured under more controlled conditions [11]. However, AEE from DLW-derived estimates (TEE minus RMR) were reported to stabilize at higher energy expenditures and—on the basis of a proposed piecewise regression model (two regression slopes with a threshold of 230 CPM/d)—it was concluded that AEE not captured by the accelerometer must have been reduced to negate the impact of AEE captured by the accelerometer. Given the magnitude of the missing AEE (∼600 kcal/d), it was proposed that this could not be due to muscular activity overlooked by the accelerometer alone but must represent a reduction in some other form of energy expenditure (for example*,* reproductive activity). Presumably, this effect must only be manifested in the AEE component because RMR was not related to TEE.

One study from hunter-gatherer children in (Shuar) is also used to directly support the constrained energy expenditure hypothesis [12]. Data for hunter-gatherer children were compared to reference data from the UK and North America. DLW was used to derive TEE (11 d), and fasting morning RMR was measured [12]. Physical activity was determined using hip-mounted accelerometry. RMR was higher in rural Shuar, and this was attributed to the energy cost of ongoing infections and immune burden based on the positive relationship between RMR and circulating Ig G concentrations [12]. Shuar children displayed little-to-no difference in TEE but lower DLW-derived AEE than industrial comparators, despite ∼25% greater accelerometry counts [12]. This was interpreted as evidence for trade-offs in childhood to constrain TEE, with the lower AEE in Shuar children possibly explained by differences in mass, efficiency, thermoregulation, or the amplitude of variation in RMR.

**FIGURE 4:**
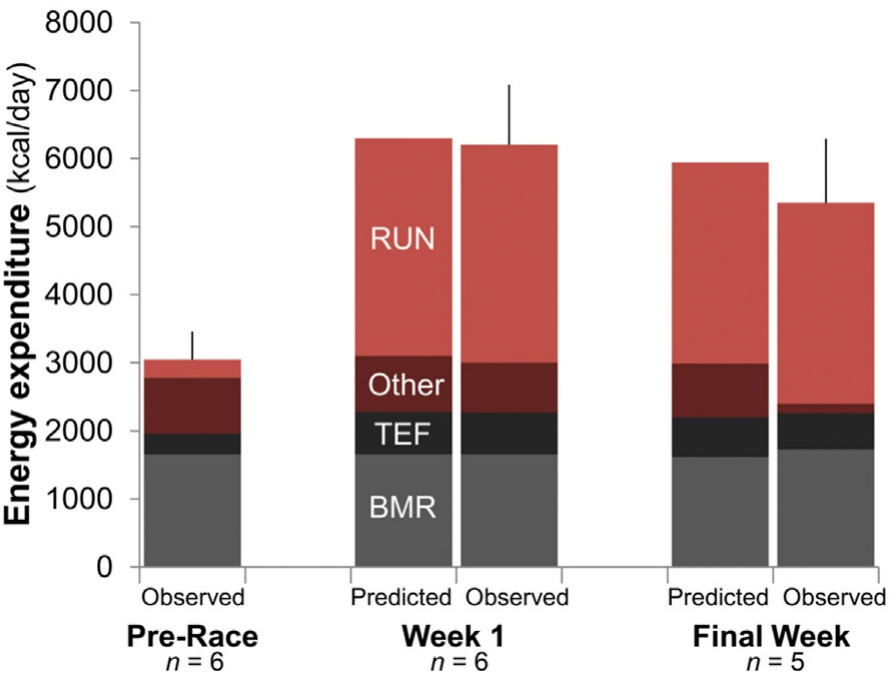
Predicted and observed components of TEE of athletes competing in the Race Across USA. Reprinted from [13]. Although at week 1, the predicted and observed components of energy expenditure appear broadly in agreement, there is a larger difference in the predicted and the observed components at week 6, primarily because of a reduction in other physical activity. BMR, basal metabolic rate; RUN, running expenditure.

Other data used to support the constrained energy expenditure hypothesis comes from a study that investigated energy expenditure in 6 adults during the transcontinental Race Across the USA, a ∼5000 km event involving running 6 d/wk for 20 wk [13]. This study incorporated measures of TEE using DLW (5 d), with running energy cost estimated using global positioning systems [13]. RMR was measured in 3 participants and estimated using predictive equations in the remaining 3. Data from the first week of the race showed strong agreement between predicted and observed energy expenditure, which increased to ∼6000 kcal/d. However, at follow-up (14 or 20 wk into the race), there was a discrepancy such that the observed TEE (from DLW) was less than predicted [13]. The predicted energy expenditure used RMR and other (nonrunning) AEE from before the race (AEE = TEE minus RMR, TEF, and running energy expenditure). These calculations indicated that it was this “other” AEE component that was less than predicted (Figure 4). There was little-to-no change in measured RMR. Thus, it was concluded that humans partially reduce components of TEE (manifested in the AEE component) [13].

**FIGURE 5:**
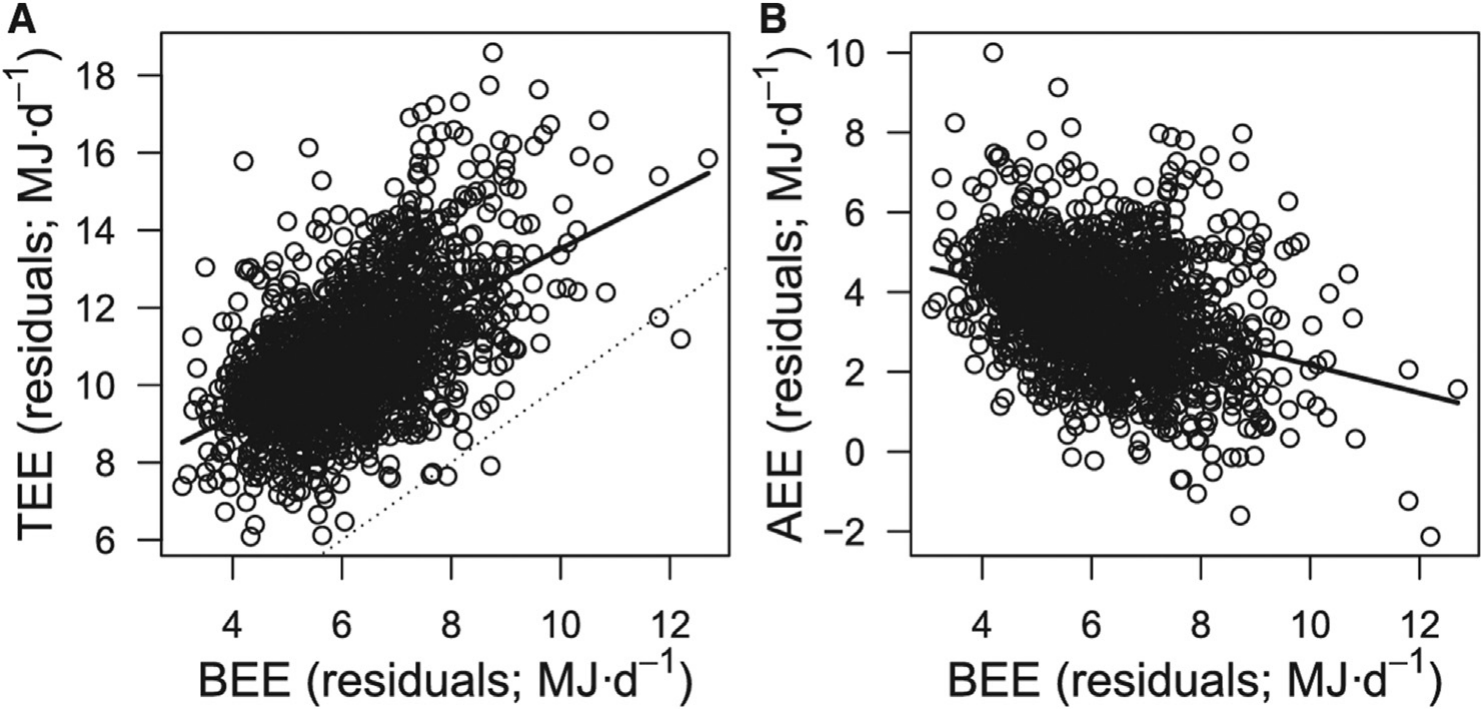
The 2 primary analyses proposed to be indicative of energy compensation. Reprinted from Careau et al. [14]. It was proposed by the authors of the article that a least squares regression slope between measured BEE and TEE (A) of <1 is indicative of compensation, and that a negative slope between measured BEE and calculated AEE (B) is also indicative of compensation. BEE, basal energy expenditure.

A final piece of recent cross-sectional evidence for the constrained hypothesis comes from an analysis of a large DLW database which included paired RMR measures from indirect calorimetry in adults (*n* = 1754) [14]. AEE was calculated by subtracting RMR from (0.9 × TEE). The primary observations used to support the constrained energy hypothesis were that the least squares regression slope for the basal energy expenditure (BEE)-TEE relationship was <1 (Figure 5A) and that the correlation between measured RMR and calculated AEE was negative (Figure 5B). The authors inferred that these relationships provide evidence of energy compensation because a lack of compensation (that is*,* an additive model) should provide a perfect positive relationship between TEE and RMR and a zero relationship between AEE and RMR [14]. To understand if compensation occurs within individuals, the authors explored within-individual relationships between residuals of RMR and of TEE for older individuals with 2 measures each and for residuals of AEE and RMR. Based on the same reasoning applied to the whole sample (relationship between RMR and TEE <1 and between AEE and RMR, negative), the authors suggested that compensation occurred within, not between, individuals [14]. The potential components that have been suggested to be responsible for the constraint across these studies, with supporting statements, are provided in Table 1.

**TABLE 1:** Summary of studies used to generate the energy constrained hypothesis, with the proposed components demonstrating constraint and supporting evidence

Type	Study	Constrained component	Supporting evidence/reasoning
Original data	Pontzer et al. (2012) [1]	BMR	TEE_ADJ_ was similar between Hadza vs. other populations a higher PAL.
	Pontzer et al. (2016) [11]	AEE	RMR_ADJ_ not different across a wide range of physical activity, assessed by accelerometry, but TEE_ADJ_ plateaued at higher accelerometry counts.
	Urlacher et al.(2019) [12]	AEE	Shuar children displayed little-to-no difference in TEE, but lower AEE vs. industrial counterparts, despite higher accelerometry counts.
	Thurber et al. (2019) [13]	AEE	Little-to-no difference in BMR, TEF or EXEE between week 1 and weeks 14/20 of an ultramarathon, but lower TEE.
	Careau et al. (2021) [14]	BMR	Relationship between BMR and TEE <1 and relationship between BMR and AEE negative.
Review	Pontzer (2015) [10]	Non-AEE metabolic activity (BMR/TEF/Other)	Cross-sectional evidence in humans and experimental data from nonhuman animals.
	Pontzer (2018) [49]	Immune function, reproduction, and stress response (BMR/TEF/Other)	Reduced markers of inflammation (*for example,* C-reactive protein) with chronic exercise, lower concentrations of sex hormones in endurance athletes, lower cortisol, and norepinephrine responses in people with high physical fitness.
	Pontzer et al. (2018) [76]	Non-AEE (BMR/TEF/Other)	Higher accelerometry counts but little-to-no differences in TEE, AEE or PAL with Hadza and Tsimane populations compared to 7 industrialized populations.
	Pontzer and McGrosky (2022) [77]	BMR	Measures of TEE at multiple timepoints indicate increase AEE is negatively associated with BMR in humans.

BMR, basal metabolic rate; EXEE, exercise energy expenditure; ADJ, adjusted for body composition and/or age.

### Critique of current evidence on constrained energy expenditure

At the simplest level, evidence from studies on energy balance components and body mass change can be used to critique the constrained hypothesis. If TEE is constant when AEE increases, then a stable body mass would require stable energy intake. Classical data from the 1950s collected from 213 Mill workers in Bengal indicated that energy intake increases by almost 1000 kcal/d in individuals performing very heavy work compared with those undertaking light work, yet body mass was reported as stable [15]. This is also supported by more recent studies on athletes [16] and nonathletes [17]. A limitation of this critique, however, is the potential inaccuracy of measuring energy intake. A more comprehensive critique requires consideration of methods of assessing energy expenditure, study design, and statistical analyses to establish appropriate inferences regarding the relationships between physical activity and other components of energy expenditure.

### Considerations for measurement of energy expenditure components

#### TEE

DLW is considered the gold-standard method of determining TEE during free-living conditions [18]. The primary principle is that labeled hydrogen disappears only from water losses, whereas labeled oxygen disappears from both water losses and exhaled carbon dioxide. Accordingly, the difference in disappearance rates of labeled oxygen and hydrogen in the body pool provides exhaled carbon dioxide over the timeframe of measurement, typically 1–3 wk [19]. TEE is obtained by estimating oxygen consumption from the measured carbon dioxide production by adjusting for the respiratory exchange ratio (RER), which is measured, assumed, or estimated. One way to estimate RER is to estimate the food quotient (FQ). When in energy balance, FQ will typically equal RER, and thus RER can be estimated from accurate food diaries. This is relevant because the diet of specific populations, such as hunter gathers, can vary substantially with regard to carbohydrate and fat content, varying both between populations, but also seasonally [20]. Because dietary intake is notoriously difficult to measure [21,22], the accuracy of estimating FQ can be challenging, ultimately impacting the accuracy of DLW estimates of TEE. Moreover, some extreme scenarios will mean that RER cannot be predicted from FQ, for example, when ketone bodies are being produced or oxidized [23]. It has been estimated that properly accounting for RER can alter the interpretation of DLW data drastically, cutting the effect size of an intervention on energy expenditure by half, from 209 ± 58 kcal/d to 104 ± 59 kcal/d [24]. Therefore, the measurement of TEE under free-living conditions is challenging, and the extra information required to accurately estimate energy expenditure is quite often likely to be missing or inaccurate from studying extreme populations.

The limitations in assessing TEE with DLW have implications for the currently available evidence on the constrained energy expenditure hypothesis. The nature of these studies often involves measures in people with vastly different body sizes, lifestyles, and diets [1] or in the same people but in very different situations, such as the phases of an ultramarathon [13]. These extreme differences could undermine or violate some assumptions of DLW for estimating TEE. Furthermore, TEE measures are normally taken without direct assessment of RER, which will reduce measurement accuracy and precision. Given these measurement uncertainties, it is risky to base interpretations on deductive reasoning and inductive reasoning using TEE measurements alone, and direct observation of the component demonstrating constraint is needed to provide greater certainty that deduced differences are not the product of measurement issues and considerations.

#### AEE

The measurement of AEE is also challenging and has implications for the energy constrained hypothesis. In some studies, AEE has been estimated by subtracting RMR (either measured or estimated) from TEE. The fact that this approach relies on 2 measures (one subtracted from another) inherently increases uncertainty compared with direct measurement (and compared to the measure of TEE) because it relies on additional assumptions and amplifies variance introduced by each measure. Moreover, without additional measurement of other components, this measure can mistakenly assign other components of energy expenditure to physical activity, such as energy costs of thermoregulation and variance in RMR across a day.

Some studies which report compensation and/or constraint have used hip-mounted accelerometers to characterize “physical activity” [11,12]. Accelerometry data (CPM/d) are used as a proxy for physical activity, with the conclusion that because higher CPM/d do not “add” to TEE, there must be compensation or constraint in some other component of energy expenditure which erodes the impact of physical activity on TEE (see Figure 3 reprinted from [11]). Although hip accelerometry is a good measure of ambulatory physical activity [25], it is notoriously poor for the assessment of nonambulatory physical activity. Hip accelerometry explains only 6 to 16% of the variance in AEE derived from DLW [[26], [27], [28]] and ∼30% of the variance in measured energy expenditure (by indirect calorimetry) during a battery of physical tasks [29]. So, at least some of the observed “constraint” could be decreases in other forms of physical activity (not detected by hip-mounted accelerometers). Hip-mounted accelerometers would not adequately capture many common forms of physical activity, such as standing, nonambulatory physical labor, load carrying, cycling, and swimming [29]. Capturing only a proportion of total activity might be useful if patterns of physical activity behavior are consistent across groups, but there is likely to be considerable heterogeneity in these types of behaviors across diverse populations [11,12]. Based on the regression shown in Figure 3, a great deal of AEE (∼600 kcal/d) is reported with zero accelerometry counts [11]. It was speculated that this could reflect other nonmuscular/movement energy expenditure allocated to AEE from DLW measurements [11], but it could simply reflect the failure of accelerometry to adequately capture the energy cost of physical activity. Thus, hip accelerometry data should not be used as a proxy for physical activity without evidence showing that this method suitably captures the nature of physical activity in a defined population, including variation due to the distinct types of representative movements undertaken in that population.

Another consideration with accelerometers is the sampling framework and recording period. Whereas DLW derives average TEE (and AEE) over a sustained period (for example*,* 5 d to 3 wk [18,19]), accelerometry data are often accepted for a given day if a device has been worn for a given proportion of the day (for example*,* 10 h or 62% [11,12]), and for a proportion of the sampling period (for example*,* 4 d [11]). Given the uncertainty in the behavior that has been missed outside the recorded period, there is a risk in trying to reconcile (fragmented) accelerometry records with summative average daily AEE data from DLW. Technical innovation and development may overcome some of these issues, for example, the integration of other physiological data to improve estimates of energy expenditure from body-mounted devices [30] and/or positioning of devices in locations that support improved wear time and sampling [31]. The accuracy of physical activity measurement is crucial for making inferences about the constrained energy hypothesis, given that this is a commonly proposed explanation for constraint (Table 1). This component needs to be measured and not deduced to make rational inferences about the relationship between physical activity and human energy expenditure.

#### TEF

The TEF is sometimes assumed in studies on the basis that fat, carbohydrates, protein, and ethanol have thermic effects of 0%–3%, 5%–10%, 20%–30%, and 12%–28%, respectively [6]. The considerable variance in TEF between macronutrients would require accurate diet assessment to derive accurate TEF, but even with accurate diet data, the range within each macronutrient is still considerable, as some variance in TEF is due to interindividual differences in the postprandial handling of nutrients, and others can be because of food form and/or degree of processing [32]. Therefore, measured TEF would be preferable, requiring ∼4 h of postprandial measures, ideally in response to a variety of foods, to understand the interactions between the individual and the foods on TEF. Studies that are aimed at investigating the constrained energy expenditure hypothesis may therefore make erroneous conclusions if TEF is estimated rather than measured directly or if the measurement is only performed in response to 1 type of meal rather than a representative mix of foods (differing in type, timing, and total amount). An erroneous conclusion could be made in either direction (that is, it is possible that constraint in TEF could be missed or that constraint is deduced when direct measurement would counter this). Studies providing evidence for the energy constrained hypothesis have often assumed TEF, which has been recognized as a limitation [11]. In the same way as AEE, TEF needs to be measured rather than assumed to provide robust and complete data on the relationship between physical activity and human energy expenditure.

#### RMR

The measurement of the lowest rate of energy expenditure (sleeping or BMR) requires participants to have fasted, asleep, in thermoneutrality, and thereby is typically assessed by room calorimetry. RMR can be assessed by either room calorimetry or indirect calorimetry when participants are awake, thereby measuring the sum of sleeping energy expenditure plus arousal. Room calorimetry is nonportable and thus is essentially never used in field studies. In these scenarios, field studies are limited to either portable indirect calorimetry devices or estimations of RMR based on prediction equations [13]. Limitations with portable metabolic systems for RMR include the inability of many devices to accurately measure ventilation rates and account for inspired gas concentrations, which can vary substantially in different environments and across time [33]. Finally, even with a single accurate estimate of RMR, there is then the assumption that this measurement reflects the full 24-h period and is stable day-to-day. A snapshot measurement of RMR is unlikely to be sufficient to extrapolate across an entire day [34,35]. Based on these limitations, evidence from a single measurement should be interpreted with caution, as they may not reflect RMR at other times of the day and/or may display some errors compared with more rigorous methods. Accordingly, measurement (rather than estimation) of RMR is required to confidently determine whether RMR is responsible for any compensation in TEE with physical activity, and multiple measures of RMR across a day are likely needed to account for circadian rhythmicity.

### Statistical issues in the interpretation of energy constraint

Alongside study design and measurement-related considerations, it is also important to consider statistical factors arising from the mainly observational studies on the constrained energy expenditure hypothesis. These potential issues include:

1) matching the statistical model with the proposed causal pathway between the exposure (independent) variable(s) and outcome (dependent) variable(s);
2) the influence of measurement error on least squares regression estimates of slope and intercept;
3) the risk of correlations being spurious because of mathematical coupling between the variables of interest;
4) the appropriate use of null hypothesis testing compared with equivalence analyses for “indistinguishable” or “no difference” type hypotheses;
5) a comprehensive and robust approach to comparing the appropriateness of nonlinear, for example, change point associations, compared with linear statistical models.

#### What are the exposure and outcome variables?

Prior to the application of any statistical model, a proposed direction of a causal pathway between the various variables of interest should be considered, preferably aided by a directed acyclic graph [36]. The causal pathway determines important aspects of the proposed statistical model [37], for example, estimates from least squares regression models can differ considerably depending upon which variable is deemed the exposure [or independent variable (x)] and the outcome [or dependent variable (y)]. The energy constraint theory indicates that increases in physical activity cause reductions in other components of energy expenditure, for example, “Increasing levels of activity may bring diminishing returns in energy expenditure because of compensatory responses in non–activity energy expenditures.” [14] (p4659). In some studies, this latter component is deemed to be in RMR. In other words, energy expended in physical activity—often using AEE derived from DLW—is the exposure (independent) variable that should be placed on the x-axis, and BEE is the outcome (dependent) variable that should be placed on the y-axis. It can be seen in Figure 5 that Careau et al. [14] selected the axes for these 2 variables in a way that is not consistent with the causal pathway for compensation theory.

It is important to select exposure and outcome in a way that is consistent with a causal pathway because this selection influences how much least squares regression estimates are affected by measurement error. Researchers should consider whether an association is “symmetric” or “asymmetric” [37]. Symmetry refers to a situation where the purpose is to estimate a slope to ultimately identify a general pattern between 2 mutually co-dependent variables [37]. If a research question is grounded in such symmetry, then least squares regression may not be appropriate for estimating a slope at all. This is because least squares regression is asymmetric so that there are 2 different lines and 2 different slopes, depending upon which variable is selected for each axis. Least squares regression is more appropriate for a definitive causal pathway between an exposure variable and an outcome variable. Along with the importance of correctly identifying exposure and outcome variables, the important issue of regression dilution is relevant to least squares regression. This issue is, in turn, dependent on the relative magnitudes of error variance between the exposure and outcome variables.

Because the energy constrained hypothesis postulates that increases in physical activity result in constrained TEE, then it follows that physical activity should, in our opinion, be the exposure on the x-axis when examining such correlations. But this is not the case in many studies.

#### Is evidence for constraint an artifact of regression dilution?

Regression dilution results when measurement errors in the predictor (x) variable attenuate the least squares regression slope [37]. The true regression slope can be 1, but measurement errors in the exposure variable (AEE) lead to the least squares regression slope being attenuated to less than one. Importantly, neither BEE nor AEE is immune from measurement errors and biological variability. Therefore, a slope of <1 as the criterion used to support the compensation hypothesis needs to be considered carefully in the context of regression dilution.

Guidelines for exploring regression dilution have been published [38], where advice is to adopt multiple approaches to diagnose and control for the effects of regression dilution, including 1) exploration of relative measurement errors between x and y variables, 2) appreciation of the causal nexus between x and y variables (see above), 3) calculation and consideration of the correlation coefficient between x and y variables (the lower the r, the more prone a least squares regression slope is to dilution, and 4) undertaking sensitivity analyses where alternative regression approaches are compared with least squares regression. It is unclear to what extent regression dilution influenced the findings of Careau et al. [14].

The use of the following published guidelines for exploring regression dilution may help to advance the understanding of whether TEE is constrained, especially given the known measurement and biological errors in components of human energy expenditure [6,18,19,39].

#### Is some evidence for constraint an artifact of spurious correlations?

Spurious correlations are those that are not explained by biological mechanisms but occur even in the absence of any biological links between correlated variables [40]. One type of spurious correlation results when a variable (x) is correlated to another variable (y), but variable x is also present in the calculation of variable y (or vice versa). In many studies, AEE has not been directly measured but, rather, has been deduced by subtracting RMR (and sometimes other estimated or measured components) from TEE [1,13,14].

**FIGURE 6:**
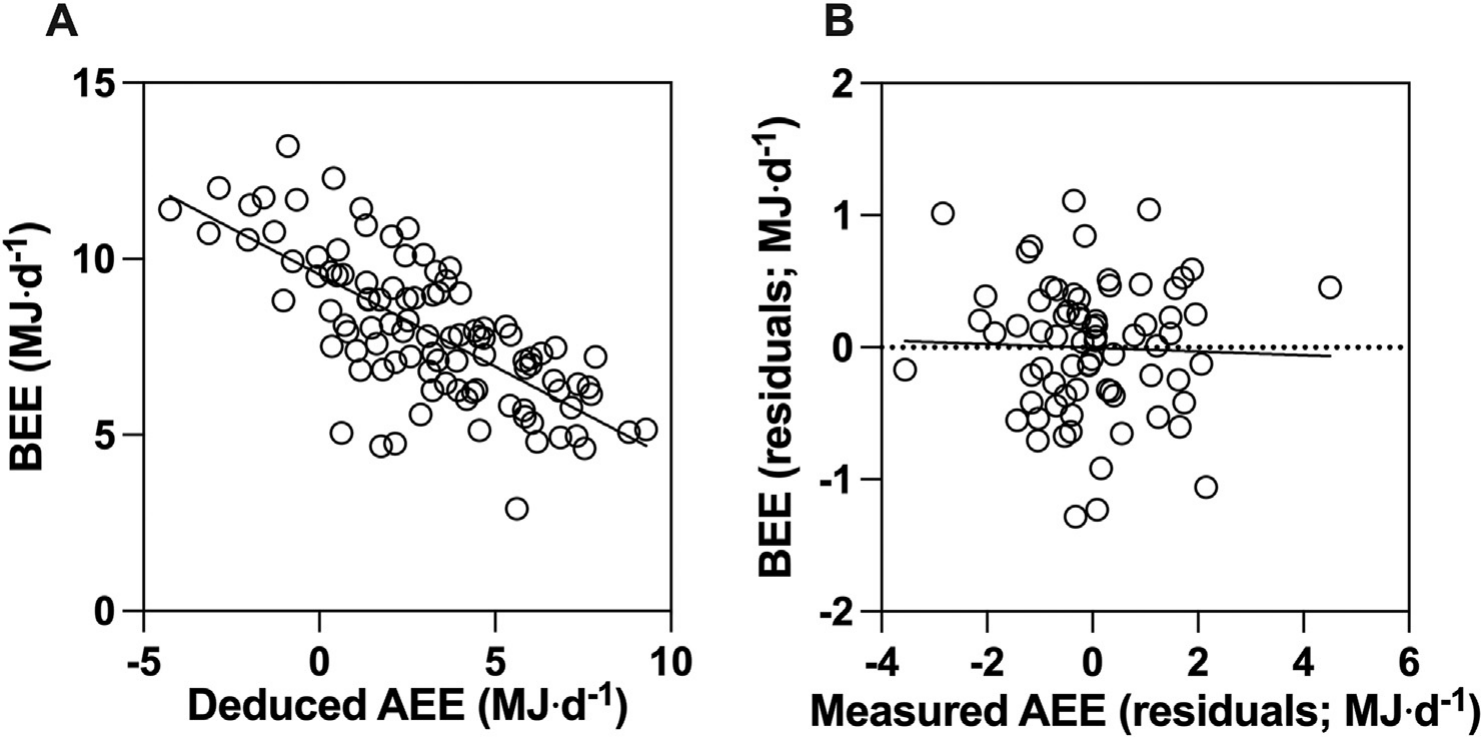
Spurious correlation between AEE and BEE (RMR), when AEE is deduced by subtracting BEE from TEE (A) and lack of correlation between measured AEE and measured BEE adjusting for covariates of sex, age, and FFM (B). Because BEE (RMR) is 1 variable, and a negative term in the calculation of AEE, the correlation shown above is entirely spurious, caused by mathematical coupling and could be present in data used to support the energy constrained hypothesis. The lack of correlation between *directly measured* AEE and BEE raises the possibility that prior reports of correlations between these measures could be because of artifacts of including the same measurement in the calculation of the variables on both the x- and y-axes. Data for panel B are from Chrzanowski-Smith et al. [41]. Fat mass was not included as a covariate in this model for 2 reasons: 1) in this dataset, FFM strongly correlated with BEE (Pearson *r* = 0.84) but fat mass shows little-to-no correlation with BEE (Pearson *r* = −0.06); 2) because the calculation of FFM and fat mass by DXA are interlinked (one is calculated by subtracting the other from total mass), the inclusion of both within a statistical model introduces the potential for multicollinearity [78]. BEE, basal energy expenditure.

AEE calculated by this subtraction method has then been correlated against RMR itself, setting up mathematical coupling and risk of spurious correlation. In Figure 6A, we present the scatterplot for the BEE-AEE correlation, whereby data have been obtained from simulation. Using the random number generator in Excel, we simulated BEE and TEE to be completely independent, uncorrelated (*r* = 0.02), and normally distributed variables (*n* = 100). Our simulation was based on mean and SD values similar to those supporting the energy constrained hypothesis [14]. Figure 6A illustrates that, in this simulation, even though BEE and TEE are separate, independent variables, a negative correlation between BEE and AEE (when AEE = TEE minus BEE) can be obtained simply because BEE is 1 of the variables in the correlation but is also a negative term in the calculation of the other variable (AEE). The correlation we present is entirely spurious, and it is unclear to what extent prior reports of constraint could be influenced by similar spurious correlations.

To reduce the likelihood of spurious correlations between AEE and RMR, such associations should ideally be explored with direct measures of each variable. If there is indeed a negative slope between these 2 measured variables, then this would support the constrained energy expenditure hypothesis. In Figure 6B, we present the scatterplot for the correlation between RMR and AEE using the data reported in a previous study where each of these variables was measured directly and independently [41]. In a similar way to Careau et al. [14], we ran a multivariable-adjusted general linear model to explore the relationship between measured BEE and measured AEE, adjusting for covariates of sex, age, and FFM.

The slope on the scatterplot in Figure 6B is −0.09 (95% CI: −0.70 to 0.52), and the correlation coefficient is 0.04. Mathematical coupling is not present in the correlation presented in Figure 6B, and the flat slope does not support the constrained energy expenditure hypothesis. There also does not appear to be any evidence for a “change point” association in the scatterplot. Incidentally, if exposure (AEE) and outcome (BEE) are reversed and remodeled (similar to Careau et al. [14]), then the slope we obtained is still flat (−0.01, 95% CI: −0.11 to 0.08). Furthermore, because our x-y and y-x slopes are very similar, then this indicates no meaningful influence of regression dilution on our least squares slope estimate [37].

Although the data we have used are from a smaller, less diverse sample, this still raises the possibility that prior correlations of DLW-derived AEE compared with BMR could be the result of including the same measurement in the calculation of variables in both the x- and y-axes.

#### Accounting for body size and composition

In some studies, the differences in body size between samples being compared are substantial, and this should be considered in order to appropriately compare measures of energy expenditure components between such groups. For example, mean body mass differed between Hadza and Western samples by ∼30 kg (∼60%–70%) [1]. It could be questioned whether the statistical models employed in comparative studies have adequately adjusted for body size and composition, especially given that: 1) body mass and energy expenditure scale allometrically; and 2) adjusting for body composition (FFM) is inherently problematic because of limitations of measurement methods.

Two common methods of assessing FFM within this field are bioelectrical impedance and dual-energy x-ray absorptiometry. However, neither of these methods can determine body cell mass, which is the most relevant measurement for RMR because cell mass is the metabolically active component of FFM. The gold-standard method for assessing cellular mass is the ^40^K dilution method. Examples of how this is relevant for normalizing RMR include evidence from energy deficits and aging. The degree of metabolic adaptation seen with severe energy deficits such as semistarvation (that is, the larger than predicted decrease in RMR seen with a recent energy deficit) can be attenuated from ∼750 kcal/d when using FFM to ∼200 kcal/d when using body cell mass (Luke and Schoeller 1992). Moreover, the apparent decline in RMR with age when adjusted for FFM is abolished when using body cell mass [42].

Accordingly, adjusting measurements of energy expenditure across populations with vastly different body size and/or composition is not straightforward, and even measures such as dual-energy x-ray absorptiometry may not be optimal for appropriate correction of body composition under extreme conditions. Including measures of body cell mass by the potassium dilution method may enhance the ability to compare TEE and TEE components across populations with large differences in body size and composition and within the same individuals before and after extreme interventions.

#### Equivalence testing compared with null hypothesis testing

Support for the compensation hypothesis often comes from the use of null hypothesis tests to conclude that the difference between 2 or more sample means is, or is not, statistically significant (*p* < 0.05). This approach is also often used for 2 or more outcomes related to energy expenditure in a differential and dichotomous fashion. For example, it has been reported that the mean PAL was greater in a sample of Hadza foragers than in a sample of Westerners, whereas it was also reported that the mean daily energy expenditure of traditional Hadza foragers was “no different” to that of Westerners [1].

It is important to highlight that a nonsignificant *p*-value from a null hypothesis test should not be used to make conclusions about the “not different” type [43]. Ironically, all a researcher would need to do to arrive at such a conclusion is recruit a small sample of participants and use outcomes that are measured with a substantial amount of random measurement error. These conditions would almost guarantee a nonsignificant p-value for a null hypothesis test on 2 sample means. For this, and other reasons, equivalence analyses have been developed specifically to arrive at conclusions regarding “no relevant difference” inferences [44,45].

For future research, various approaches are available for equivalence analyses; a common approach involves “2 1-sided tests.” In this frequentist interval approach, the null and alternative hypotheses within each set are reversed. Equivalence is concluded only if both 1-sided tests statistically reject the presence of effects equal to or larger than a threshold value that is deemed to be clinically or practically relevant. This approach places informed thresholds for minimal clinically important differences (MCID) at the center of the inferential process. Without such an MCID, a statistically significant difference may be negligible, or a nonstatistically significant difference could be important. There have been very few efforts to arrive at a consensus regarding MCIDs in exercise science, despite the recent publication of formal and informed methods [46]. Importantly, the difference in a study outcome might not be statistically significant merely because it is associated with more measurement error than another study outcome that has been found to be statistically significantly different.

Our primary point here is that conclusions of “no statistically significant difference” are commonly used in components of research on energy compensation, yet informed and robust indications of MCIDs seem absent in the field, raising the likelihood that important differences between samples are not being detected because of the emphasis on null hypothesis testing, alongside issues of small samples and differential amounts of measurement error between study outcomes. We also believe that this field of research would benefit from careful consideration of directional (one-sided) or nondirectional (two-sided) null hypothesis tests when such testing is appropriate, for example, for testing whether the mean BMR of 1 sample is specifically larger than another sample.

#### Comparison of linear and nonlinear models

**FIGURE 7:**
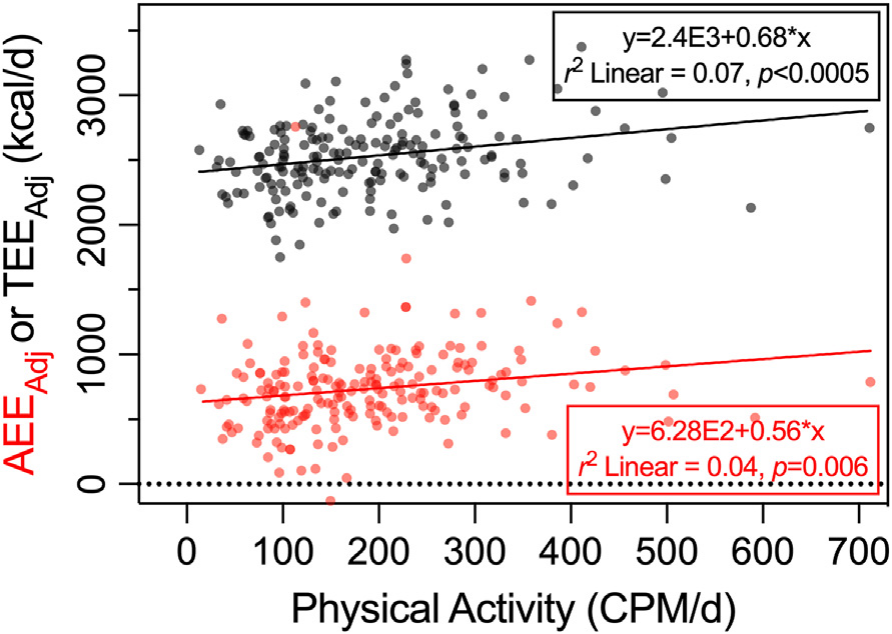
Data extracted from Figure 3A in Pontzer et al. [11]. In this report, a fitted 2 piecewise regression slopes to these data (below and above 230 CPM/d). In the present figure, linear regression slopes were fitted and demonstrate a good fit with TEE_Adj_ and AEE_Adj_. AEE_adj_, adjusted AEE; TEE_adj_, adjusted TEE.

Pontzer et al. [11] proposed that TEE and AEE varied in a nonlinear fashion when plotted against accelerometry counts. After various explorations with different set values, they proposed a cut-off or “change point” threshold of 230 CPM/d and applied piecewise (segmented) regression to suggest that a linear model was appropriate when physical activity was below this threshold. For physical activity higher than this threshold, it was suggested that the regression slope is zero, that is, the association “stabilizes.” There was no formal model comparison in arriving at this claim of nonlinear (stabilization at a higher physical activity) associations between physical activity and TEE or AEE. Ideally, information would be provided to show that the selected piecewise nonlinear model provides a “better” fit than a single linear model across the whole measurement range of physical activity. Although some model comparison procedures were reported to be employed by Pontzer et al. [12], other more modern statistical criteria, such as Akaike’s information criterion, can also be used to inform any comparison of the relative fit of 2 competing statistical models [47]. The relevant question is whether a single linear model for the data in scatterplots presented by Pontzer et al. [11] can be ruled out in preference of a piecewise nonlinear model. Using the Digitizeit software, we extracted the adjusted AEE and TEE data from Figure 3 in Pontzer et al. [11]. Using these data, it is debatable whether a piecewise nonlinear model is a more appropriate fit to the data than the single linear model we fitted (Figure 7). The coefficient of determination of 0.06 (6%) for this single linear model is higher than the 2 piecewise models reported to fit the data by Pontzer et al. [11] and is statistically significant (*P* < 0.0005). Regression model selection is crucial for the interpretation of some key data supporting the energy constrained hypothesis. It is unclear whether linear or nonlinear models best fit the currently available data. Future studies should explore this choice objectively, alongside other relevant considerations such as allometric scaling [48].

### Biological plausibility and potential mechanisms underlying constraint

**FIGURE 8:**
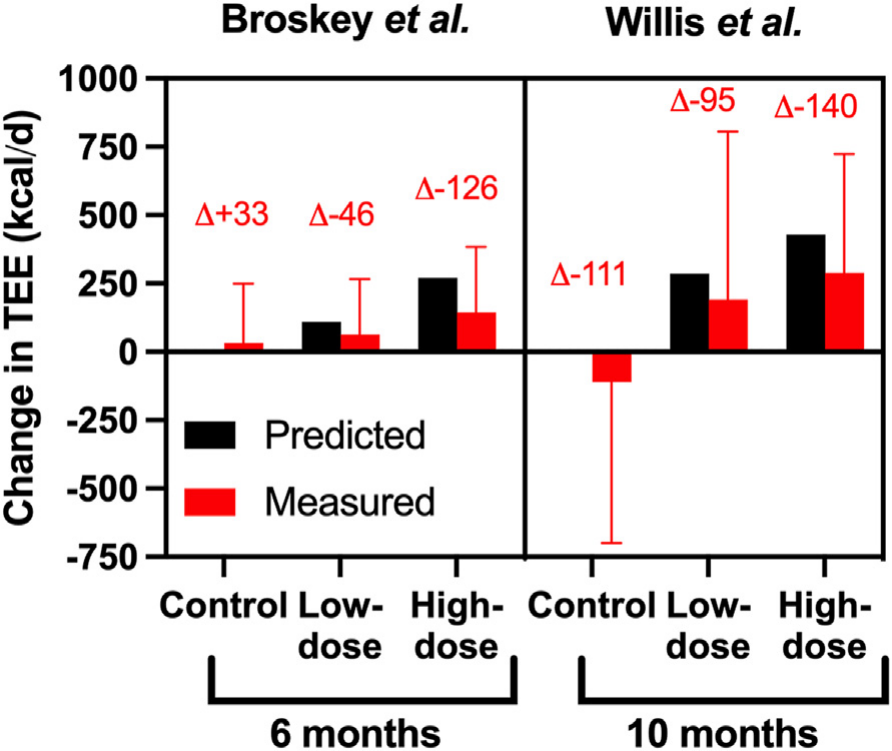
Predicted and measured changes in TEE from 2 randomized controlled trials of increasing exercise on TEE [50,51]. Each demonstrate some evidence for compensation because the measured increases in TEE are less than the predicted increases. Delta values represent the difference between predicted and measures TEE. Data are means ± SD.

As discussed above, the evidence from empirical studies in humans often used to support the constrained energy expenditure hypothesis is underdeveloped, and more empirical data are needed with additional considerations of measurement and statistical approaches to confirm or refute this hypothesis. However, the evolutionary argument for energy expenditure compensation and constraint under conditions of increased TEE is persuasive [10,49]. Furthermore, nonhuman animal studies indicate constraint of TEE with increased physical activity across a variety of birds and mice in tightly controlled experiments [10]. There is also some evidence supporting some degree of compensation from 2 long-term (6–10 mo) randomized controlled trials (RCTs) of exercise training in specific populations of adults with DLW estimates of energy expenditure [50,51]. These trials were not designed to determine compensation, and whereas both show that prescribed exercise > 200 kcal/d will lead to an increase in TEE, the effect is less than predicted (50%–66% on average), and there appears to be some form of compensation (Figure 8). Although it should be noted that—at least in 1 study—the magnitude of this difference between predicted and observed TEE was similar in the control group compared with the exercise groups, suggesting that the observation of a mismatch between predicted and observed TEE could be expected for several reasons other than constraint because of increased physical activity (for example, trial effects, seasonal effects, measurement errors, etc.). The less-than-predicted weight loss with exercise interventions has often been attributed to dietary compensation [52,53], but these 2 RCTs with DLW measures of TEE indicate that at least part of the explanation may involve less-than-predicted changes to energy expenditure [50,51]. The biologically plausible mechanisms underlying the less-than-predicted changes to energy expenditure with supervised exercise from these RCTs and other relevant studies will now be discussed.

#### RMR

The mean changes in morning RMR reported in the 2 long-term RCTs ranged between −50 to +40 kcal/d [50,51]. This is consistent with the wider literature, with meta-analysis revealing the difference in RMR with aerobic exercise training is +82 kcal/d (95% CI: −58, 221). Therefore, evidence from RCTs does not support a reduction in RMR with increased exercise, even in studies that indicate some form of compensation for TEE. Thus, gross effects on RMR are unlikely to be a major (single) mechanism underlying compensatory reductions in energy expenditure leading to constraint in TEE.

If the circadian fluctuations in RMR across the day were attenuated with high levels of physical activity, DLW estimates of AEE would incorrectly allocate the decrease in TEE to AEE rather than RMR if RMR is only taken as a morning snapshot. This is likely to only exert a modest effect because the amplitude in the circadian variation of RMR is ∼55 kcal/d [34]. Nevertheless, to accurately quantify all components of TEE, studies are needed to directly assess RMR at different times of the day and at low and high levels of physical activity, ideally within individuals and at different states of energy (im)balance.

#### NEAT

NEAT is a substantial and highly malleable subcomponent of TEE. Even within the confines of a chamber respirometer, with no scheduled physical activity, NEAT is ∼400 kcal/d on average in a large sample of adults and can be as high as 800 kcal/d [54]. These activities comprise miscellaneous and often incidental physical activity, including a diverse range of movements such as fidgeting, play, standing, mastication, and self-care [55]. In free-living nonexercizers with an average PAL, NEAT could easily be ∼800–1000 kcal/d because of the energy cost associated with other tasks such as occupation, household chores, and childcare [7,56]. From an evolutionary perspective, it might be sensible to “cut back” on the nonessential components of NEAT before making other metabolic and physiological changes. This could involve some conscious decisions (for example, choosing to drive rather than walk because of a perceived exercise “credit”). In humans, feeding and fasting appear to influence NEAT within just a few days [57,58]. Thus, NEAT is a large component of energy expenditure that is biologically regulated, and differences in NEAT could account for compensation in TEE.

In 1 of the long-term RCTs that indicates some form of compensation [51], data from room calorimetry indicated a reduction in spontaneous physical activity (NEAT) under chamber conditions, suggesting compensation in physical activity may have contributed to the lower-than-predicted TEE. There was no evidence for this effect from hip-mounted accelerometry data under free-living conditions in either trial [50,51], but this could reflect the limited ability of this technique to capture AEE (discussed in section 2.1.2). Other chamber-measured components of energy expenditures (SMR, arousal, and TEF) did not account for the less-than-expected increase in TEE [51]. In the ultramarathon Race Across the USA, the reduction in observed TEE (“other” AEE) was likely explained by reductions in NEAT (Figure 4) [13].

The idea that increases in exercise can lead to less-than-expected increases in TEE because of compensation and substitution of other physical activity is not new [59]. If the compensation of NEAT accounts for the observed constraint, then this would have very different implications than if the constraint occurred in a nonbehavioral component of energy expenditure because the behavioral components can be (at least theoretically) more directly manipulated to counteract or prevent compensation and constraint. In future studies, better measures of NEAT are required to examine whether this explains the apparent compensation in exercise training studies.

#### Physical activity efficiency

The degree of coupling between internal to external work is often termed exercise efficiency. Changes in efficiency would not be detected by accelerometry and would appear as reductions in AEE if AEE is estimated by RMR minus TEE using DLW. The mechanisms that underpin efficiency include biomechanical, biochemical, and physiological components and could be altered by physical activity status, providing a potential mechanism for apparent constraint.

Differences in gait can have a profound impact on exercise efficiency [60]. Because gait and other movement patterns could be altered by repeatedly performing specific movement patterns, it is possible that humans find the most efficient movement pattern with repeated practice, resulting in a lower energy cost for that activity. Biochemical aspects are primarily related to the fuels oxidized during physical activity; because the oxidation of fat requires more oxygen for the equivalent energy expended than does the oxidation of carbohydrate [23], people on a high carbohydrate diet display a gross efficiency during cycling of ∼20.4% compared with 19.6% on a lower carbohydrate diet [61]. Finally, there are physiological aspects, such as muscle mitochondrial efficiency, that also contribute to exercise efficiency [62]. Importantly, human muscle mitochondrial efficiency has been demonstrated to increase following high-intensity interval training [63], suggesting a possible mechanism by which prolonged increases in physical activity may decrease the energy cost of movement.

It is plausible that with long-term increases in physical activity, adaptations relating to increased efficiency occur, which uncouple measured energy expenditure from the expected increase in energy expenditure, supporting a constrained model. Without measuring efficiency of movement under differing levels of physical activity and across a wide range of tasks representative of daily physical activity, differences in efficiency could cloud inferences regarding the nature of any compensation or constraint.

#### Altered TEF

Changes in TEF could underlie apparent energy constraint in several ways. First, even if the diet is similar, TEF could decrease with high PALs. Cross-sectional evidence supporting this includes lower TEF in endurance-trained athletes compared to controls in response to a meal providing 10 kcal/kg FFM (∼56 kcal/180 min compared with 79 kcal/180 min) [64]. However, even if TEF is reduced by high physical activity, it is questionable whether the magnitude is meaningful for TEE, as an extrapolation of this difference to 4 meals across a day equates to a difference of <100 kcal/d. It is possible, however, that constraint in TEE exists as the sum of very small decreases in energy expenditure within multiple components, with the cumulative total being meaningful. Second, in response to increases in physical activity, people may change the amount and composition of their diet, which in turn, would alter TEF directly (as discussed in section 2.1.3) and/or potentially via changes in the gut microbiome [65].

#### Is energy balance rather than energy expenditure the signal?

Energy expenditure should not be considered in isolation because there are important interactions between components of energy intake and energy balance which consequently affect energy expenditure. When in an energy deficit, RMR can decrease greater than would be predicted by the loss of FFM [66]. This phenomenon is called metabolic adaptation (or adaptive thermogenesis). This phenomenon is relatively short-lived and responds in the reverse direction, where RMR increases with energy surplus [67]. When people increase physical activity to very high levels, it is possible that energy intake does not match expenditure, and thus an energy deficit is created, thereby (transiently) reducing RMR and producing apparent constraint. For the energy constrained model to substantially change understanding, it would need to refer to physical activity-induced changes in metabolism that occur independent of energy imbalance because energy deficit-induced adaptive thermogenesis is already a relatively well-established phenomenon. Although the 2 RCTs discussed in section 3 [50,51] demonstrated no meaningful or statistically significant effects on RMR, this does not rule out the possibility that participants could have been in a brief period of energy balance prior to the postintervention RMR measurement, and if the RMR measures had been taken at another time, perhaps when participants were in an energy deficit, RMR might have been lower. Accordingly, whereas recognizing the difficulty in this amount of control in humans, the state of energy (im)balance should be considered at each measurement point in future studies of both cross-sectional and interventional nature.

Further support for the idea of energy deficit driving reductions in TEE comes from evidence that metabolic signals such as 3,5,3′-triiodothyronine and testosterone decrease with energy deficit, but not with energy surplus, even in the face of sustained high energy expenditure equating to 4000–4250 kcal/d [68]. Indeed, recent data provide further support for this, demonstrating that people in energy balance or energy surplus display TEE-activity responses consistent with the additive model, whereas individuals in an energy deficit display TEE-activity responses consistent with the constrained model [28].

This energy deficit hypothesis fits well with evolutionary and physiological viewpoints. Increased physical activity threatens energy balance, and energy deficits threaten survival in resource-limited environments. Therefore, from an evolutionary perspective, it is likely that energy deficit is the causal link rather than physical activity per se. Physiologically, hormonal changes with energy deficits, such as reductions in leptin concentrations, can cause conservation of energy-consuming physiological processes such as menstruation. Correction of hypoleptinemia with recombinant leptin can improve reproductive function in women with low body weight or high physical activity and hypothalamic amenorrhea [69]. Furthermore, decreases in leptin correlate with metabolic adaptation [70], and leptin replacement can prevent the decline in RMR following an energy deficit [71]. Therefore, energy deficit and consequent changes in hormonal concentrations could result in constrained TEE via reductions in RMR.

Energy (im)balance has also been shown to influence NEAT [55], whereby NEAT decreases during energy deficit by as much as ∼300 kcal/d and increases during energy surplus by a mean of ∼300 kcal/d [72,73]. Substantial interindividual variability in this response is also observed, whereby increases in NEAT with a surplus of 1000 kcal/d ranged from negligible to >700 kcal/d. This variation is clearly meaningful for energy balance because it explained the majority of variance (*r* = 0.77) in fat gain during a 1000 kcal/d surplus [73]. This highlights the importance of direct measurement of NEAT to capture the full potential for compensation and constraint in energy expenditure under differing degrees of energy (im)balance.

It could be expected that the largest effect of adaptive thermogenesis would be in the most extreme energy deficits over the longest periods of time. As an indication of the degree of energy deficit to which the constrained model is plausible, the Minnesota starvation experiment restricted energy intake to ∼50% of baseline intake for 6 mo. The reduction in RMR adjusted for FFM was ∼400 kcal/d [74]. Therefore, it is conceivable that this reduction might represent the maximum effect of adaptive thermogenesis, and it would take extreme reductions in RMR (greater than those with 6 mo of semistarvation) to offset increases in AEE of >400 kcal/d.

### Solutions and Future Directions

Based on current evidence, there is insufficient evidence to fully support either the additive or the constrained model of human energy expenditure. Most data to date are from cross-sectional observations and statistical models comparing populations with extreme differences in a variety of characteristics, which may negatively impact measurements. Some are based on deductive inferences rather than direct measurement or studies lacking a suitable control group. In addition, the only RCTs of exercise training with DLW measures of TEE were not directly designed to measure compensation, and many outcomes are still deduced rather than measured or measured as snapshots and under specific conditions, potentially missing variation across a day or within different conditions. The compensatory reduction has not yet been directly demonstrated and thus is derived from deductive inference. There is, therefore, a need for adequately powered, long-term, RCTs with gold-standard methods that directly quantify the major components of energy expenditure to assess if human energy expenditure is constrained or additive and to identify the source and nature of the compensation and constraint.

There is little evidence to support the extreme constrained model proposed as:

*“The bottom line is that your daily (physical) activity level has almost no bearing on the number of calories that you burn each day” (p103)* [2].

An upper limit of TEE probably exists [75], but this is likely irrelevant for most people, and large changes in physical activity will alter TEE. Indeed, ultramarathon studies such as the Race Across the USA study supports the *additive* model more than the constrained model, as there was a huge increase in TEE (+2500 kcal/d) even after 20 wk [13]. Therefore, even if some constraint exists, it is unlikely to fully offset physical activity, such that further increases in physical activity will result in a net increase in energy expenditure, just not in a linear manner.

Measurements of energy expenditure components are imperfect, and variation can never be eliminated, which means that deduction cannot be used to establish where constraint may exist in energy expenditure. To overcome these limitations, triangulation of methods could be employed, with measurements repeated at multiple time points and under varying dietary and environmental conditions to capture the full circadian, energy balance, and lifestyle conditions that could modulate any compensation and constraint.

These controlled trials could be combined with statistical models to account properly for changes in body size and composition. The appropriate statistical approach might include a noninferiority analysis with a justifiable margin of noninferiority between expected (based on the increase in AEE) and observed TEE defined a priori. Direct measurement of the component that is expected to demonstrate constraint is required. This is important for several reasons, including the simple notion that we may not fully appreciate all components of energy expenditure that could demonstrate constraint, although it could be that constraint manifests in small changes in each component, summing across multiple components to produce a meaningful reduction in the expected TEE.

Although there is a key need to collect more data to establish which model of energy expenditure is closer to truth, currently available data indicate that neither the simple additive nor the extreme constrained models (that is*,* where physical activity adds nothing to TEE) are likely to be correct, and the true response likely resides somewhere in between. In energy balance, large increases in physical activity will add to and increase TEE, but the effect appears to be less than predicted. The less-than-expected increase in TEE when energy balance is maintained could be due to increased mitochondrial efficiency, increased efficiency of force transfer across the muscle-tendon unit, more efficient movement patterns, or other factors such as compensatory behaviors and reductions in nonexercize activity thermogenesis. RCTs are needed to address these questions, with multiple designs to test the different contexts, such as energy balance and energy deficit.

## Data Availability

All data are previously published or available in the main text.
